# Nitrogen deficiency results in changes to cell wall composition of sorghum seedlings

**DOI:** 10.1038/s41598-021-02570-y

**Published:** 2021-12-02

**Authors:** Reza Ramdan Rivai, Takuji Miyamoto, Tatsuya Awano, Rie Takada, Yuki Tobimatsu, Toshiaki Umezawa, Masaru Kobayashi

**Affiliations:** 1 Division of Applied Life Sciences, Graduate School of Agriculture, Kyoto University, Kyoto, 606-8502 Japan; 2 Indonesian Institute of Sciences, Bogor, 16003 Indonesia; 3 Research Institute for Sustainable Humanosphere, Kyoto University, Uji, Kyoto, 611-0011 Japan; 4 Division of Forest and Biomaterials Science, Graduate School of Agriculture, Kyoto University, Kyoto, 606-8502 Japan; 5 Present Address: Sakeology Center, Niigata University, Ikarashi, Niigata 950-2181 Japan

**Keywords:** Abiotic, Plant physiology, Solid biofuels, Secondary metabolism, Cell wall

## Abstract

Sorghum [*Sorghum bicolor* (L.) Moench] has been gaining attention as a feedstock for biomass energy production. While it is obvious that nitrogen (N) supply significantly affects sorghum growth and biomass accumulation, our knowledge is still limited regarding the effect of N on the biomass quality of sorghum, such as the contents and structures of lignin and other cell wall components. Therefore, in this study, we investigated the effects of N supply on the structure and composition of sorghum cell walls. The cell walls of hydroponically cultured sorghum seedlings grown under sufficient or deficient N conditions were analyzed using chemical, two-dimensional nuclear magnetic resonance, gene expression, and immunohistochemical methods. We found that the level of N supply considerably affected the cell wall structure and composition of sorghum seedlings. Limitation of N led to a decrease in the syringyl/guaiacyl lignin unit ratio and an increase in the amount and alteration of tissue distribution of several hemicelluloses, including mixed linkage (1 → 3), (1 → 4)-β-d-glucan, and arabinoxylan. At least some of these cell wall alterations could be associated with changes in gene expression. Nitrogen status is thus one of the factors affecting the cell wall properties of sorghum seedlings.

## Introduction

A transition from fossil fuels to renewable energy has been considered the key to attaining energy security and environmental sustainability. As of 2018, renewable energy accounted for approximately 14% of the global primary energy supply^1^, and this share is expected to grow further^2^. Bioenergy is a sector of renewable energy that includes municipal waste, industrial waste, solid biofuels, biogases, and liquid biofuels, and it accounts for approximately 67% of the current renewable energy mix^1^. Hence, increased production of bioenergy feedstocks would be significant to facilitate the shift to renewable energy, in turn, stimulating the demand for biomass as the feedstock for bioenergy.

Sorghum [*Sorghum bicolor* (L.) Moench] is a multi-purpose crop that delivers food, fodder, and fibers^3–5^. Recently, it has received increasing attention as a raw material for bioenergy^6–13^. The strategy for converting sorghum into energy depends on the type of sorghum^7^. Grain sorghum produces starchy grains that can be a cost-effective source of starch used in ethanol production^12^. Sweet sorghum accumulates sugar in its stalk, which can also be utilized for fermentation. In addition, biomass sorghum can produce more than 42 t ha^−1^ biomass under favorable conditions^11^, which can be utilized for solid biofuel applications such as biopellets or biochar^7–10^. Another advantage of sorghum is its adaptability to adverse conditions, such as drought or salinity^14^. This characteristic makes it possible to grow sorghum on land that is not suitable for the cultivation of other crops such as rice or maize, as a way to produce biomass feedstock without encroaching on existing arable lands used for food production.

One constraint for crop production in marginal lands is low soil fertility. Among the plant nutrients taken up from the soil, nitrogen (N) is required in the greatest quantities and hence often becomes a growth-limiting factor. The impact of N supply on sorghum biomass accumulation has been well documented^15–19^. However, information on the effect of N deficiency on the quality of sorghum biomass is limited. The quality of biomass as a feedstock for energy production depends on its composition. Grass cell walls are mainly composed of cellulose, hemicelluloses, and lignin. Cellulose, the crystalline aggregate of (1 → 4)-β-d-glucans, is the most abundant component of grass cell walls. Hemicelluloses are associated with cellulose as crosslinks to form the cellulose-hemicellulose network as the framework of the cell walls. Major hemicelluloses in grasses include non-crystalline (1 → 4)-β-d-glucans, mixed linkage (1 → 3), (1 → 4)-β-d-glucan (MLG), arabinoxylan, glucuronoxylan, and xyloglucan^20^. Lignin is a phenylpropanoid polymer that fortifies cell walls by filling up the spaces between the polysaccharides^21–23^ and is biosynthesized via oxidative coupling of *p*-hydroxycinnamyl alcohols (monolignols) and related compounds formed in the cinnamate/monolignol pathway^24,25^. Lignin is composed of three major units: syringyl (S), guaiacyl (G), and *p*-hydroxyphenyl (H), which are formed by the polymerization of sinapyl, coniferyl, and *p*-coumaryl alcohols, respectively^24,25^. Grass lignin, especially in S units, is often acylated with *p*-coumarate^21,26,27^. Moreover, a flavone, tricin, has been found to serve as a lignin monomer in grasses^26,28–30^. Ferulate is also abundant in grass cell walls and is thought to serve as a crosslink among hemicelluloses and between lignin and hemicelluloses^31^.

In general, lignin content is positively correlated with the heating value of lignocellulose^32^; therefore increased lignin content can be an advantage when the cell wall, or bagasse, is considered as a source of solid biofuels ^13,23,30,33^. On the other hand, higher lignin content can lead to higher cell wall recalcitrance against enzymatic digestion^21^. Hence, it is important to understand the effects of N nutrition on lignin and other cell wall components in biomass crops. There have been several reports on the impact of N supply on cell wall composition in grasses. For example, lignin content was decreased by N fertilizer application in rice (*Oryza sativa*)^34^, maize (*Zea mays*)^35^, and brachypodium (*Brachypodium distachyon*)^36^. By contrast, N fertilizers increased the lignin content in cell walls of giant miscanthus (*Miscanthus* × *giganteus*)^37^. These findings suggest that the effect of N-supply on cell wall components differs among plant species. In sorghum, a recent report^38^ has shown that application of N increased the lignin content in sweet sorghum cultivated in a semi-arid environment. However, it remains to be investigated precisely how each cell wall component can be affected by N supply, and by what mechanism the change is induced in sorghum. Therefore, in this study, we investigated N deficiency-induced changes in cell walls of sorghum seedlings, with the aim of determining early effects of different N status on sorghum cell wall structure. The study was conducted using a hydroponic culture system to minimize the intervention by other factors, and the properties of cell walls from N-deficient plants and those from plants receiving sufficient amounts of N were compared through chemical, two-dimensional (2D) heteronuclear single quantum coherence (HSQC) nuclear magnetic resonance (NMR), gene expression, and immunohistochemical analyses.

## Results

### Chemical analyses of cell walls

**Figure 1:**
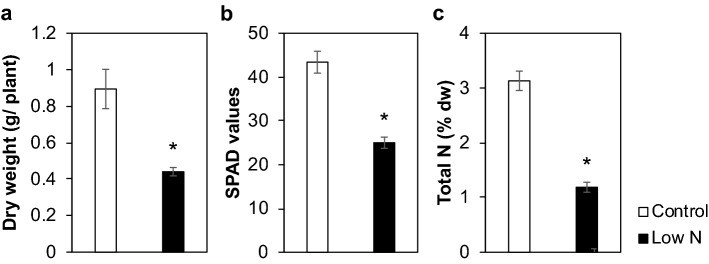
Effect of low-N treatment on hydroponically-grown sorghum seedlings growth. (**a**) Dry weight, (**b**) soil plant analysis development (SPAD) values, and (**c**) N content of hydroponically-grown sorghum seedlings cultivated under control or low-N conditions at 3 weeks after treatment. Values are means ± SD (n = 3). Asterisks indicate significant differences between control and low-N plants. (Student’s *t* test, *p* < 0.05).

Sorghum seedlings were grown hydroponically using a standard culture medium and a medium containing a low level of N (1/10th), as control and low-N treatments, respectively. The low-N treatment reduced the dry weight of seedlings by 51% (Fig. 1a), with decreased chlorophyll and N contents (Fig. 1b,c). The impact of low-N treatment on cell wall properties was then examined by chemical analyses. Thioglycolic acid lignin analysis suggested that N supply did not significantly affect lignin content (Table 1). However, the lignin aromatic composition was affected by N supply, as there was a 27% decrease in the thioacidolysis-derived syringyl/guaiacyl-type monomer ratio (S/G unit ratio) in low-N plants, with a 48% increase in the G-type monomer composition, as compared to the control plants (Table 1). The contents of cell wall-bound hydroxycinnamates, including ferulic acid (FA) and *p*-coumaric acid (*p*CA), were also analyzed. The content of cell wall-bound *p*CA, which is mainly attached to lignin^39,40^, did not differ between control and low-N plants. On the other hand, the content of FA, which is mainly attached to arabinoxylan ^27,40–42^, was increased by 56% under low-N conditions (Table 1).

**Table 1:** Cell wall chemical analysis of hydroponically-grown sorghum seedlings cultivated under control and low-N conditions at 3 weeks after treatment. Asterisks indicate significant differences between low-N and control plants (Student’s *t* test, *p* < 0.05). CWR: cell wall residue, H: *p*-hydroxyphenyl, G: guaiacyl, S: syringyl, *p*CA: *p*-coumarate, and FA: ferulate, TFA: trifluoroacetic acid. Values are means ± SD (n = 3).

	Control	Low N
**Lignin content**		
Thioglycolic acid lignin (mg g^−1^ CWR)	100.8 ± 4.6	103.5 ± 9.0
**Lignin composition by thioacidolysis**		
H (µmol g^−1^ CWR)	1.7 ± 0.1	2.0 ± 0.1*
G (µmol g^−1^ CWR)	32.0 ± 0.9	45.7 ± 2.1*
S (µmol g^−1^ CWR)	13.1 ± 1.3	13.9 ± 1.6
(H + G + S) (µmol g^−1^ CWR)	46.7 ± 1.3	61.7 ± 3.6*
H (%)	3.5 ± 0.3	3.3 ± 0.3
G (%)	68.5 ± 1.9	74.2 ± 1.1*
S (%)	28.0 ± 2.3	22.5 ± 1.3*
S/G ratio	0.41 ± 0.0	0.30 ± 0.0*
**Cell wall-bound hydroxycinnamates**		
Saponified hydroxycinnamate yield		
*p*CA (mg g^−1^ CWR)	9.7 ± 0.7	9.6 ± 0.9
FA (mg g^−1^ CWR)	3.4 ± 0.4	5.3 ± 0.1*
**Polysaccharides**		
TFA-insoluble fraction (from crystalline cellulose)		
Glucose (mg g^−1^ CWR)	208.9 ± 23.6	248.9 ± 26.1
TFA-soluble fraction (from matrix polysaccharides)		
Arabinose (mg g^−1^ CWR)	33.2 ± 1.9	50.5 ± 2.7*
Xylose (mg g^−1^ CWR)	91.2 ± 8.6	128.0 ± 4.9*
Galactose (mg g^−1^ CWR)	11.8 ± 0.4	17.5 ± 0.2*
Glucose (mg g^−1^ CWR)	27.1 ± 1.2	58.3 ± 2.5*
Mannose (mg g^−1^ CWR)	5.4 ± 0.8	6.0 ± 0.5
Uronic acid (mg g^−1^ CWR)	70.8 ± 1.9	57.8 ± 6.4*
**Mineral**		
Calcium (mg g^−1^ CWR)	8.7 ± 0.5	4.8 ± 0.3*

Regarding the polysaccharide fraction, the content of crystalline cellulose was higher in low-N plants, but the difference was not statistically significant (Table 1). Analysis of the glycosyl residue composition of the trifluoroacetic acid (TFA)-soluble, non-crystalline polysaccharide fraction indicated that the low-N plants contained more arabinosyl, xylosyl, galactosyl, and glucosyl residues than the control plants (Table 1). The glucosyl residue content increased more than twofold upon low-N treatment. We also estimated changes in pectins, another class of cell wall polysaccharides, by quantifying uronic acids, and calcium (Ca) possibly bound to pectin. The uronic acid and Ca contents were decreased by 18% and 45% in low-N plants, respectively (Table 1).

Methylation analysis revealed the presence of at least 16 glycosyl residues with different linkages in the cell walls of sorghum seedlings (Table 2). Of the sugar residues listed in Table 1, mannose, which was probably derived from mannans, remained unaffected by the low-N treatment. Therefore, we estimated the amount of each detected residue relative to 4-linked mannosyl. The glycosyl residues that were increased by low-N treatment included 3- or 4-linked glucosyl and 4- or 3,4-linked xylosyl residues (Table 2). In grass cell walls, the 3-linked glucosyl residue is found in callose [(1 → 3)-β-d-glucan] and MLG, whereas the 4-linked glucosyl residue in non-crystalline (1 → 4)-β-d-glucan and MLG. The 4- and 3,4-linked xylosyl residues are found in xylans with or without substitution, such as xylan, arabinoxylan, or glucuronoxylan. The observed increase of these residues suggests that the cell walls of low-N plants contained more hemicelluloses and/or callose. Galactosyl residues were found to be increased in the composition analysis (Table 1) but not in the methylation analysis (Table 2) of the glycosyl residue. The reason for this apparent discrepancy is unclear at present.

**Table 2:** Glycosyl linkage composition of the cell walls of hydroponically-grown sorghum seedlings cultivated under control and low-N conditions at 3 weeks after treatment. Relative abundance of the residues was calculated as molar ratio relative to 4-linked mannosyl residue. The averages of duplicate (control) or triplicate (low-N) determinations are shown. Values are the average of two (control) or three (low N) replicate samples.

Residue	Position of *O*-CH_3_ group	Deduced linkage	Relative abundance	
			Control	Low N
Arabinose	3,5	2-linked furanose	0.35	0.33
	2,5	3-linked furanose	0.59	0.62
	2,3	4-linked pyranose	1.53	1.65
Xylose	2,3,4	terminal	0.77	1.08
	2,3	4-linked pyranose	5.35	8.46
	2	3,4-linked pyranose	2.53	3.67
Galactose	2,3,4,6	terminal	1.17	0.86
	3,4	2,6-linked	0.37	0.15
	2,4	3,6-linked	0.96	0.88
	2,3	4,6-linked	2.31	2.08
Glucose	2,3,4,6	terminal	2.29	4.51
	2,4,6	3-linked	0.94	1.11
	2,3,6	4-linked	13.13	21.47
Mannose	2,3,4,6	terminal	0.43	0.39
	2,3,6	4-linked	1.00	1.00
	2,6	3,4-linked	0.81	0.84

### Nuclear magnetic resonance analysis

**Figure 2:**
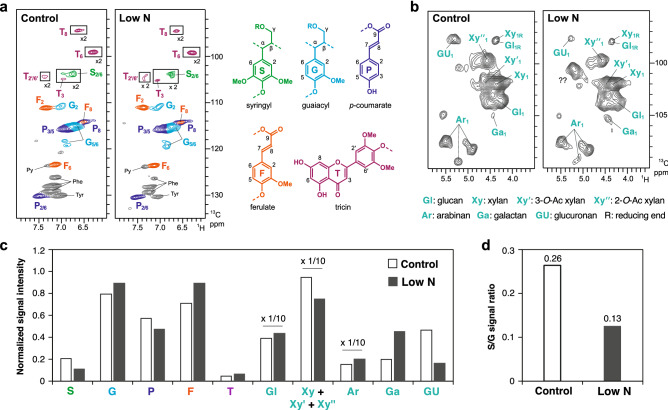
Two-dimensional short-range ^1^H-^13^C correlation nuclear magnetic resonance (2D HSQC NMR) spectra of the cell walls of hydroponically-grown sorghum seedlings cultivated under control and low-N conditions at 3 weeks after treatment. The NMR spectra were acquired with composite samples prepared from three replicates. (**a**) Aromatic sub-regions showing signals from major lignin hydroxycinnamate aromatic units. Contours are color-coded to match the displayed structures. Boxes labeled × 2 means regions with scale vertically enlarged twofold. (**b**) Anomeric sub-regions showing signals from major cell wall polysaccharide units. Py, pyridine (solvent). Phe and Tyr, phenylalanine and tyrosine residues in residual proteins^45^. (**c**) Normalized intensity of major lignin, hydroxycinnamate and polysaccharide signals expressed on a **S** + **G** = 1 basis. Data labeled × 1/10 indicate that the reported values are divided by a factor 10 for visualization purposes. (**d**) S/G signal ratio.

To further investigate the low N-induced alterations of the sorghum cell wall structure, we performed 2D HSQC NMR analysis on the cell wall samples from the sorghum seedlings using the NMR approach^43,44^. The aromatic sub-regions of the obtained HSQC NMR spectra displayed well-resolved contour signals from the lignin aromatic units such as S (**S**), G (**G**), and tricin (**T**) units, along with the signals from the hydroxycinnamate FA (**F**) and *p*CA (**P**) units (Fig. 2a). In addition, the sugar anomeric sub-regions of the spectra displayed contour signals from cell wall polysaccharide components, including glucan (**Gl**), non-acetylated (**Xy**), and acetylated (**Xyʹ** and **Xyʹʹ**) xylan, arabinan (**Ar**), galactan (**Ga**), and glucuronan (**GU**) units (Fig. 2b). To estimate the structural differences between the control and low-N sorghum cell walls, these contour signals were integrated and normalized based on the sum of the S and G lignin aromatic signals (**S** + **G**) (Fig. 2c,d).

The S (**S**) and G (**G**) lignin signals were relatively decreased and increased, respectively, in the HSQC spectrum of the low-N sorghum cell walls compared to those in the HSQC spectra of the control cell walls (Fig. 2c). Consequently, the **S**/**G** signal ratio was notably reduced in the low-N cell wall spectrum (0.13) compared to that in the control cell wall spectrum (0.26) (Fig. 2d). This result was in accordance with the significant reduction in the S/G monomer ratio, as determined by thioacidolysis (Table 1). In addition, the FA (**F**) signals were notably increased in the low-N cell wall spectrum, corroborating the chemical analysis data that showed that cell-wall-bound FA released by mild-alkaline hydrolysis was increased in the low-N sorghum cell walls (Table 1). In terms of the polysaccharide components, the normalized intensities of glucan (**Gl**), arabinan (**Ar**) and galactan (**Ga**) signals were all higher in the low-N cell wall spectrum compared to those in the control cell wall spectrum (Fig. 2c). Overall, these results are in agreement with the results of the chemical analysis (Tables 1, 2). The low-N cell wall spectrum also displayed reduced glucuronan (**GU**) signals (Fig. 2c), supporting the decreased uronic acid content in the low-N cell walls as determined by chemical analysis (Table 1). On the other hand, the xylan signals (sum of the non-acetylated and acetylated xylan signals, **Xy** + **Xyʹ** + **Xyʹʹ**) appeared to be lower in the low-N cell wall spectrum than in the control cell wall spectrum (Fig. 2c), which is in contrast to the results of the chemical analysis, which showed increased xylan in the low-N cell walls (Tables 1, 2). The reason for this discrepancy is unclear.

### Expression of cell wall-related genes

**Figure 3:**
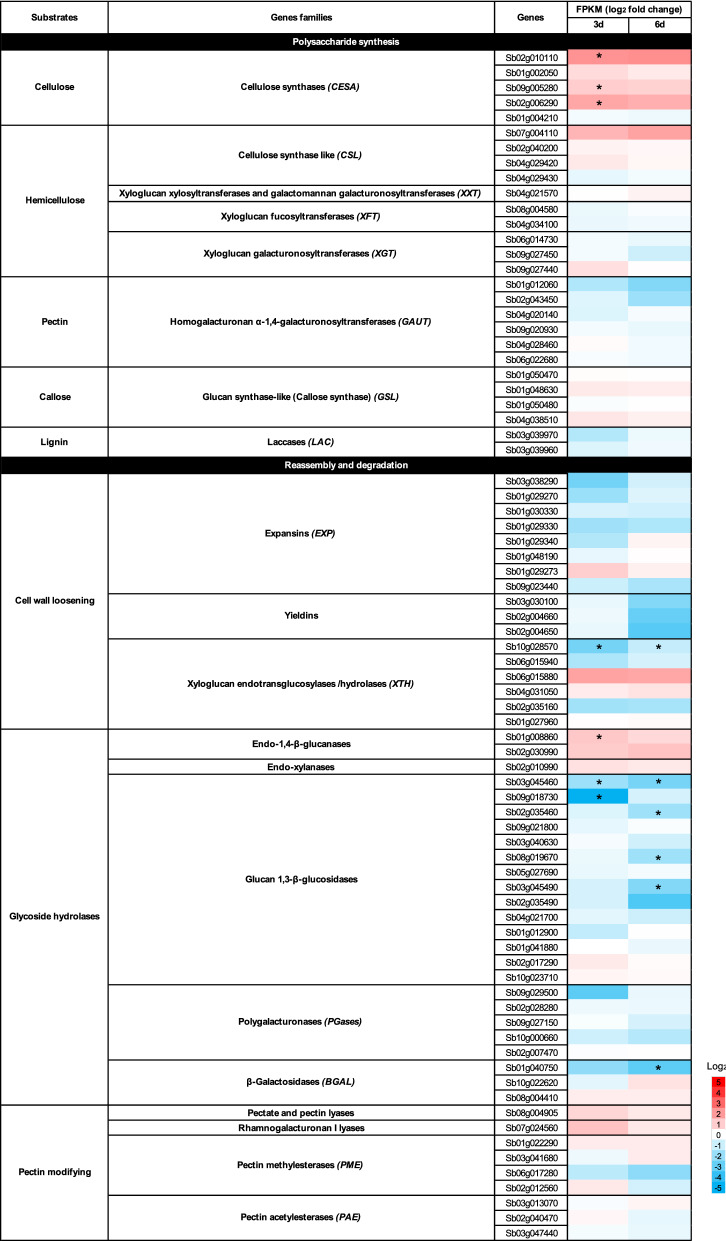
Heatmap showing the change in expression of known cell wall-related genes in hydroponically-grown sorghum seedlings in response to low-N condition at 3 and 6 days after treatment. The log_2_ values of the fold change (low-N/control) are shown. Asterisks indicate significant difference between treatments (n = 3, *q*-value < 0.05).

To obtain insights into the mechanisms underlying the N limitation-induced changes in sorghum cell walls, we conducted gene expression analyses. Sorghum genes related to cell wall biosynthesis and modification were selected according to a previous report^46^, and their expression under low-N conditions was investigated by referring to data from our previous RNA-sequencing analysis of N-deficient sorghum seedlings^47^. Of the genes examined, the *endo-1,4-β-glucanase* (Sb01g008860) encodes a homolog of Arabidopsis KORRIGAN 1 (KOR1), which plays an essential role in cellulose biosynthesis as an integral part of the cellulose synthase complex^48^. Hence, although the gene has been categorized as a glycosyl hydrolase in the list (Fig. 3), it is more likely to be involved in cellulose biosynthesis rather than glycan degradation. Three days after initiating the low-N treatment, expression of the genes encoding cellulose synthase (CESA) and endo-1,4-β-glucanase was upregulated in low-N plants, whereas expression of two genes encoding glucan 1,3-β-glucosidase, an enzyme degrading callose, was downregulated (Fig. 3). Expression of other glucan-related genes, including *cellulose synthase-like proteins* (*CSL*) and *glucan synthase-like proteins* (*GSL*), were not statistically different between low N and control plants (Fig. 3). A similar tendency was observed with the expression of these genes at 6 d, but the statistically significant difference was not demonstrated between control and low-N plants (Fig. 3).

A gene encoding xyloglucan endotransglucosylase/hydrolase (XTH) was downregulated in low-N plants, while the expression of *endo-xylanase* was not modulated significantly (Fig. 3). As for the expression of pectin-related genes, a gene for β-galactosidases (BGAL), which possibly catalyzes the degradation of rhamnogalacturonan I (RG-I) side chains, was downregulated by low N treatment at 6 d (Fig. 3). Meanwhile, no significant change was observed in other pectin-related genes, including *homogalacturonan α-1,4-galacturonosyltransferases* (*GAUT*), *polygalacturonases* (*PGases*), *pectate and pectin lyase*, *RG-I lyase*, *pectin methylesterase*, and *pectin acetylesterase* (Fig. 3). Expression of *laccase* (*LAC*), which may be involved in lignin monomer polymerization, was not significantly modulated by low-N treatment (Fig. 3).

**Figure 4:**
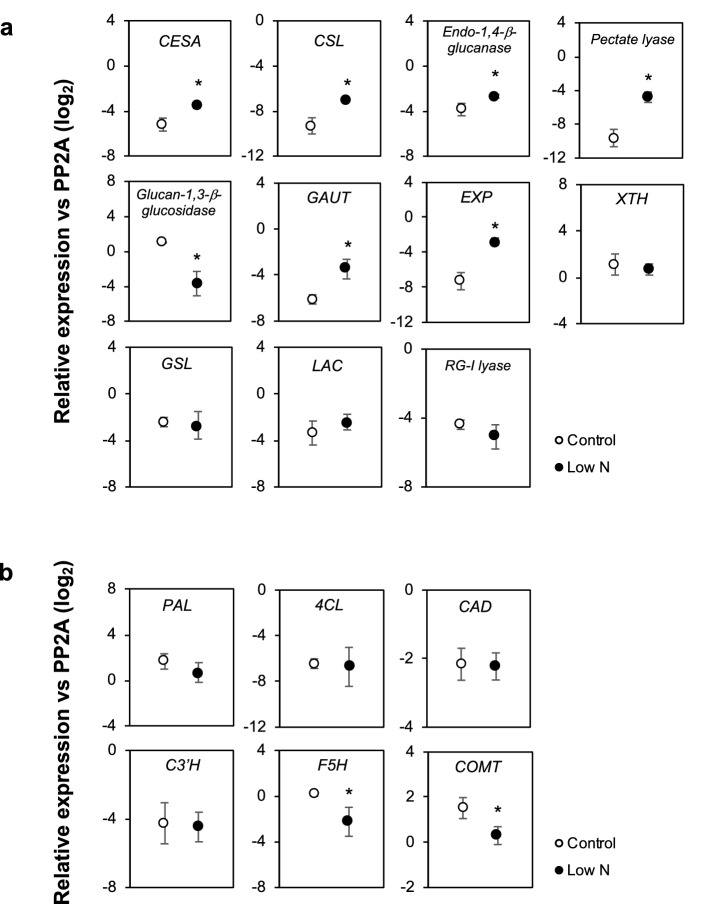
Reverse transcription-quantitative PCR analysis of the expression of selected cell wall-related genes in low nitrogen (N)-treated hydroponically-grown sorghum seedlings. The analysis was conducted at 3 weeks after treatment. (**a**) Polysaccharide metabolism-related genes, and (**b**) lignin biosynthesis-related genes. Genes subjected to the analysis have been listed in Supplementary Table S1. *CESA*: *Cellulose synthase A*, *CSL*: *cellulose synthase-like*, *GAUT*: *homogalacturonan α-1,4-galacturonosyltransferase*, *EXP*: *expansin*, *XTH*: *xyloglucan endotransglucosylase/hydrolase*, *GSL*: *glucan synthase-like/callose synthase*, *LAC*: *laccase*, *RG-I*: *rhamnogalacturonan I*, *PAL*: *phenylalanine ammonia-lyase*, *4CL*: *4-coumarate CoA ligase*/*Brown midrib 2* (*Bmr2*), *CAD*: *cinnamyl alcohol dehydrogenase*/*Brown midrib 6* (*Bmr6*), *C3ʹH*: p-*coumaroyl ester 3-hydroxylase*, *F5H*: *ferulate 5-hydroxylase* (= *coniferaldehyde 5-hydroxylase*, *CAld5H*), and *COMT*: *caffeate/5-hydroxyferulate* O*-methyltransferase* (= *5-hydroxyconiferaldehyde* O*-methyltransferase*, *CAldOMT*)/*Brown midrib 12* (*Bmr12*). The expression of each gene was analyzed as transcript abundance relative to *PP2A* (XM_002453490). Values are means ± SD (n = 3). Asterisks indicate significant differences between control and low-N plants. (Student’s *t* test, *p* < 0.05).

The above analysis suggested altered expression of cell wall-related genes in sorghum seedlings at 3 or 6 d after low-N treatment. We then further assessed the impact of N limitation on cell wall-related gene expression at 3 weeks after treatment, using a reverse transcription-quantitative PCR (RT-qPCR) analysis. The target genes chosen were those showing the largest change (either up- or down-regulated under low N supply) in each group (Fig. 3). As shown in Fig. 4a, the analysis confirmed the upregulated expression of *CESA* and *endo-1,4-β-glucanase* under low-N conditions. Furthermore, the analysis demonstrated upregulation of *CSL*, *pectate lyase*, *GAUT*, and *expansin* (*EXP*) and downregulation of *glucan-1,3-β-glucosidase* in low-N plants (Fig. 4a), although their expression was not changed significantly at 3 or 6 d as revealed by RNA sequencing analysis (Fig. 3). Statistically significant changes were not observed with the other targets, including *XTH*, *GSL*, *LAC*, and *RG-I lyase* (Fig. 4a). These results confirmed the effect of low N condition on cell wall components in hydroponically-grown sorghum seedlings.

Several known genes involved in lignin biosynthesis were also included in the analysis (Fig. 4b). Of these, *phenylalanine ammonia lyase* (*PAL*)^49,50^, *4-coumarate CoA ligase* (*4CL*)/*Brown midrib 2* (*Bmr2*)^51,52^, *cinnamyl alcohol dehydrogenase* (*CAD*)/*Brown midrib 6* (*Bmr6*)^51,53–56^, and p*-coumaroyl ester 3-hydroxylase* (*C3ʹH*)^52^ did not show significant changes following the low-N treatment (Fig. 4b). On the other hand, two genes associated with S-lignin biosynthesis, that is, *ferulate 5-hydroxylase* (*F5H*) (= *coniferaldehyde 5-hydroxylase*, *CAld5H*)^49,57,58^ and *caffeate/5-hydroxyferulate* O*-methyltransferase* (*COMT*) (= *5-hydroxyconiferaldehyde* O-*methyltransferase*, *CAldOMT*)/*Brown midrib 12* (*Bmr12*)^51,59,60^ were downregulated in low-N plants (Fig. 4b).

### Immunohistochemical analyses

**Figure 5:**
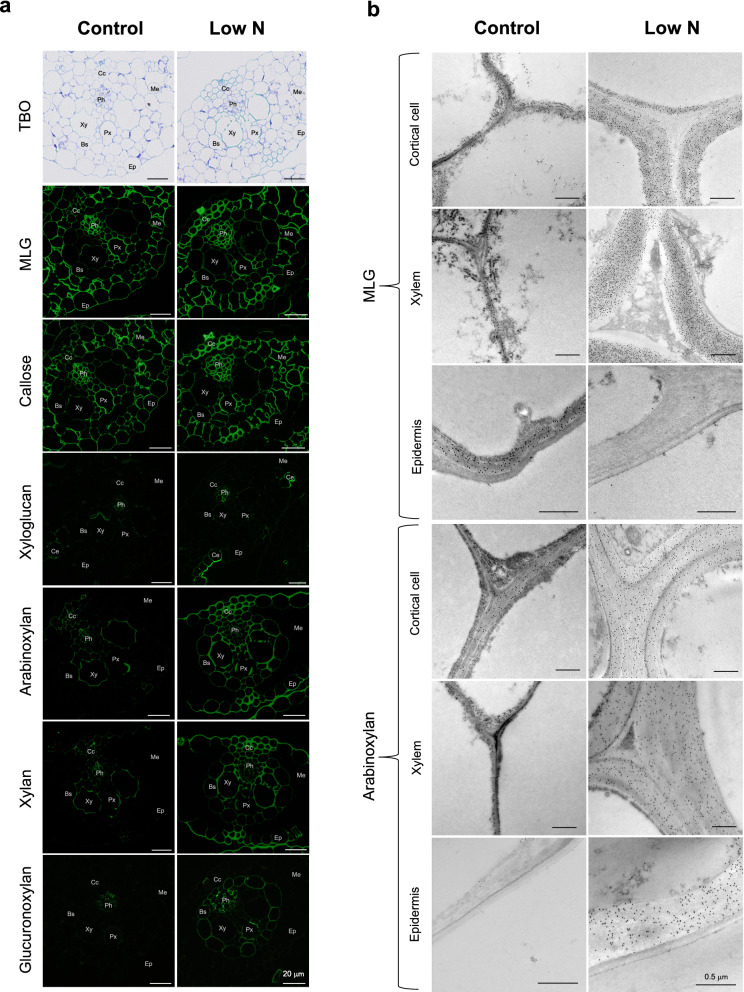
Immunohistochemical analyses of hydroponically-grown sorghum seedlings cultivated under control or low-N conditions at 3 weeks after treatment. (**a**) Toluidine blue O (TBO) staining and immunofluorescent labeling using anti-mixed-linkage (1 → 3), (1 → 4)-β-d-glucan (MLG), (1 → 3)-β-d-glucan (callose), xyloglucan, arabinoxylan, xylan, and glucuronoxylan antibodies, and (**b**) immunogold labeling using anti-MLG and arabinoxylan antibodies. Ep: epidermal tissues, Ce: curved epidermal tissues, Cc: cortical cells, Me: mesophyll tissues, Bs: bundle sheath, Xy: xylem, Px: protoxylem, Ph: phloem.

The abundance and localization of several glucans and xylans were examined by immunostaining. In sections from the aboveground part of sorghum seedlings, which mainly consisted of developing leaf sheath and leaf blade tissues, fluorescence signals from anti-MLG antibody and anti-(1 → 3)-β-d-glucan (callose) antibody were distributed in all parts of the section, including epidermal, mesophyll, cortical, and vascular tissues (Fig. 5a). These signals from both anti-MLG and callose antibodies in cortical sclerenchyma were more intense in low-N plants than in control plants. Conversely, the signals in epidermal tissues were lower in low-N plants than in control plants (Fig. 5a). Signals from anti-xyloglucan antibody (LM15) were present only in phloem and curved epidermal tissues and were stronger in low-N plants than in control plants (Fig. 5a). The fluorescence signals from the antibodies recognizing arabinoxylan (LM11) and xylan (LM10) occurred specifically in epidermal tissues and vascular bundles, and the signals were more intense in low-N plants than in control plants (Fig. 5a). These signals were also detected in epidermal cells in low-N plants, but not in the control plants (Fig. 5a). Mesophyll cells were not stained by antibodies in either low-N or control plants. The fluorescence signal from anti-glucuronoxylan antibody (LM28) was present in vascular bundles, excluding cortical sclerenchyma cells, but not in epidermal or mesophyll tissues in both low-N and control plants. Glucuronoxylan signals in vascular bundles were more intense in low-N plants than in control plants (Fig. 5a). Taken together, these results suggest that the amounts and/or distribution of several glucans and xylans were modified under low-N conditions.

**Figure 6:**
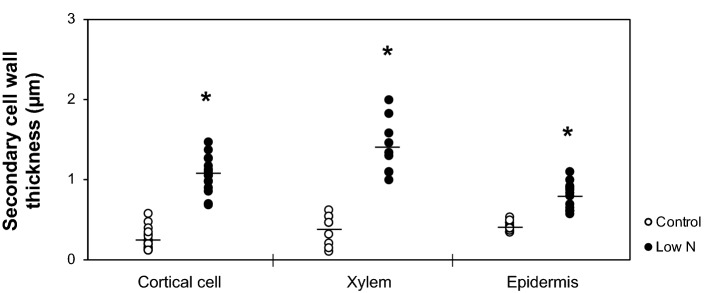
Cell wall thicknesses of cortical, xylem, and epidermal cells of hydroponically-grown sorghum seedlings cultivated under control or low-N conditions at 3 weeks after treatment. Points indicate each measured value; 18 measurements from 6 individual cells for cortical cells, 9 measurements from 3 individual cells for xylem, and 12 measurements from 4 individual cells for epidermal cells. Horizontal bars indicate the averages. Asterisks indicate significant differences between controls and low-N plants (Student’s *t* test, *p* < 0.05).

Because MLG and arabinoxylan are the two most abundant hemicellulosic polysaccharides in cell walls of young, elongating organs of grasses^61^, we investigated their abundance and distribution in low-N and control seedlings using immunoelectron microscopy. With the anti-MLG antibody, the labeling in the cortical cell and xylem walls was more abundant in low-N plants than in control plants. In contrast, essentially no labeling was observed in the secondary cell walls of low-N plant epidermal cells, whereas abundant labeling was found in the corresponding region of control plants (Fig. 5b). With the anti-arabinoxylan antibody, cortical cell and xylem walls in low-N plants were labeled more heavily than those of control plants (Fig. 5b). The difference in labeling abundance was more apparent in the secondary cell walls of the epidermal tissues (Fig. 5b). In addition, the secondary cell walls of cortical, xylem, and epidermal tissues appeared to be thicker in low-N plants than in control plants (Fig. 5b). We then estimated the cell wall thickness using image analysis. The results confirmed that the secondary cell walls in these tissues were significantly thicker in the low-N plants (Fig. 6).

## Discussion

In this study, we demonstrated that N deficiency affects the structure and properties of cell walls of hydroponically-grown sorghum seedlings through a series of cell wall structural analyses. First, N deficiency can affect the composition and structure of cell wall lignin. The total yield of the thioacidolysis-derived lignin monomers was significantly higher in the low-N plant cell walls than in the control plant cell walls (Table 1). A similar result was previously reported for maize grown under low N supply^35^. However, the lignin content estimated by the thioglycolic acid method did not differ significantly between the low-N and control plants (Table 1), suggesting that the increased yield of the thioacidolysis-derived lignin monomers from the low-N plant cell walls might reflect an increase in the frequency of lignin β–*O*–4 linkages, which are specifically cleaved by thioacidolysis to release quantifiable lignin monomers^62^, rather than an increase in the total lignin content. On the other hand, our thioacidolysis and 2D HSQC NMR analyses both showed that the S/G unit ratio of the lignin polymer in the low-N plant cell walls was reduced compared to that in the control plant cell walls (Table 1, Fig. 2). As β–*O*–4 linkages are typically more abundant in S-lignin units than in G lignin units, such reductions in S/G unit ratio can decrease the frequency of β–*O*–4 linkages in lignin polymers^63,64^. However, our data are in contrast to this notion. Further investigations on the chemical structures of lignin in sorghum are needed to clarify this aspect.

Nitrogen limitation might cause a shift in the monolignol biosynthesis in favor of the G-type monomer rather than the S-type monomer. Such responses might be explained by the reduced availability of *S*-adenosylmethionine, which is synthesized from methionine and used as the methyl donor in the conversion of G-type precursors to S-type precursors^65^. However, the direction of change in S/G ratio induced by low N varies among reports; in contrast to the decreased S/G ratio observed for hydroponically-grown sorghum seedlings in this study, the S/G ratio was notably increased in poplar^66^, *Eucalyptus*^67^, and maize^35^. The specific response of the S/G ratio to N deficiency may vary depending on species and conditions, such as the severity of deficiency. Such a modification may arise at least partly through transcriptional changes of the genes in monolignol biosynthesis. In the current study the expression of *F5H* (*CAld5H*) and *COMT* (*CAldOMT*)/*Bmr12*, the genes required for S-lignin synthesis, were significantly downregulated in low-N plants (Fig. 4b), while the other genes tested (*PAL, 4CL*/*Bmr2*, *CAD*/*Bmr6*, and *C3ʹH*) were not significantly changed (Fig. 4b).

The limitation of N also affected the polysaccharide moiety in the cell walls of hydroponically-grown sorghum seedlings. The low-N plant cell wall contained more hemicellulosic glucans and xylans, as shown by chemical, NMR, and immunohistochemical analyses (Table 1, Figs. 2,5). In line with this finding, the content of cell wall-bound FA, mainly attached to arabinoxylan^27,40–42^, was also substantially increased under low-N conditions (Table 1, Fig. 2). Electron microscopy analysis also showed that the cell walls of low-N plants were thicker than those of control plants (Fig. 6), probably due to the enhanced formation of secondary cell walls. A previous study also reported enhanced cell wall thickening in 5-week-old sorghum seedlings grown in soil under N-limited conditions, particularly in bundle sheath cells^68^. Our current results showed that low N-induced cell wall thickening also occurred in tissues other than the bundle sheath, including cortical, xylem, and epidermal tissues (Fig. 6). The observed increase in the amounts of hemicelluloses might be due in part to enhanced secondary cell wall formation, as hemicelluloses are one of the major components of secondary cell walls. We also found specific increase of arabinoxylan, as suggested by the denser labeling of secondary cell walls by anti-arabinoxylan antibody in immunoelectron microscopy (Fig. 5b).

At least a part of the change in polysaccharide content was a result of differences in expression of genes for the synthesis and/or degradation of these polysaccharides, as determined by RNA-sequencing and RT-qPCR (Figs. 3,4a). The upregulated expression of *CESA*, *CSL*, and *endo-1,4-β-glucanase* (Fig. 3,4a) could contribute to enhanced synthesis of glucans, including non-crystalline 1,4-β-d-glucan and MLG. Conversely, the downregulated expression of *glucan 1,3-β-glucosidase* (Fig. 3,4a) might have led to the over-accumulation of callose in low-N plants. Notably, these changes in cell wall-related gene expression occurred as early as 3 days after the start of low-N treatment (Fig. 3), suggesting that alteration of cell wall components could be an adaptation strategy for N starvation. Under insufficient N supply, plants limit the synthesis of N-containing molecules, such as proteins and amino acids. This metabolic adjustment reduces the demand for carbon skeletons for N assimilation. The surplus carbon may be accumulated as starch or utilized for cell wall materials, as previously reported in maize leaves^69^. Hoch (2007)^70^ previously suggested that hemicelluloses in the cell wall are not only structural components but also mobile carbon stores. The increase in glucans and xylans observed in the current study might also be a sink for surplus carbon. Possibly consistent with the findings of this study, it was previously shown that an increased N availability reduced the content of hemicelluloses in soil-grown giant miscanthus^37^.

Effect of N limitation on pectin has been previously reported, for example, the amount of pectin was reduced under low-N conditions in grapevine callus^71^. The form of N supply also affected the pectin content and structure in the hydroponically-cultured brachypodium cell wall^36^. In the current study, uronic acid content decreased under low-N conditions (Table 1). However, we did not determine whether the observed decrease was in galacturonic acid residues in pectin or glucuronic acid residues in hemicelluloses. The 2D NMR analysis suggested a decrease in glucuronic acid residues in low-N plant cell walls (Fig. 2), but because NMR reveals only relative amounts between treatments, it remains unknown how much of the detected decrease in uronic acid could be explained by that of glucuronic acid. The result of gene expression analysis was also complicated, as it showed an upregulated expression of both *GAUT* and *pectate lyase* as the enzymes for pectin synthesis and degradation, respectively (Fig. 4a). Thus, in the current study, it is difficult to conclude whether pectin was decreased by N limitation in hydroponically-cultured sorghum seedlings. Further study is necessary to clarify this aspect.

Since the composition of cell wall varies significantly depending on plant age and environmental conditions, the findings of this study using hydroponically-cultured seedlings may not be directly applicable to field-grown, mature plants. Nonetheless, the results demonstrate that the N nutrition status is one of the factors affecting the properties of sorghum cell wall, by affecting the expression of relevant genes (Figs. 3,4). The properties affected include S/G lignin unit ratio. Because the presence of methoxyl groups leads to a lower percentage of carbon in monolignols, the decrease of S-lignin over G-lignin under limited N supply may lead to an increase in the heating value of the material^23,72^. The change in S/G lignin unit ratio may also affect cell wall degradability, although the relationship between S/G lignin unit ratio and degradability somehow varies among reports depending on the grass biomass source and biomass treatment technology^72–75^. In this study, we investigated the effects of N deficiency alone, but the effect of excess N on sorghum cell wall properties should also be examined in future studies. In addition, the form of N applied can be another factor that may affect sorghum cell wall properties, as shown with effects on the hemicellulose content in brachypodium^36^. Further studies may contribute to a better understanding of the relationship between the N nutritional condition and cell wall properties of sorghum plants.

## Methods

### Plant materials and growth conditions

We used sorghum (*S. bicolor*) cv. BTx623 as a well-characterized and genome-sequenced cultivar. The seedlings were cultivated hydroponically in a culture room and subjected to low-N treatment, as previously described^47^. The medium for the control condition was Yoshida B culture solution containing 0.25 mM (NH_4_)_2_ HPO_4_ and 1 mM Ca (NO_3_)_2_. The medium for the low-N treatment contained N at a concentration 1/10th that of the control (0.05 mM NH_4_^+^ and 0.2 mM NO_3_^−^). The concentrations of calcium and potassium were maintained at the same level as that of the control using CaCl_2_ and KH_2_PO_4_, respectively. A completely randomized design with three replications was used in this experiment. The cultivation was carried out at Kyoto University, Kyoto, Japan. Our study on plants complied with relevant institutional, national, and international guidelines and legislation.

### Chlorophyll and nitrogen content analyses

Chlorophyll content was measured in the third youngest fully expanded leaves, as previously described^47^, using the Soil Plant Analysis Development (SPAD)-502 plus chlorophyll meter (Konica Minolta, Tokyo, Japan). The aerial parts of the 6-week-old (3 weeks after low-N treatments) seedlings were dried in an oven at 70 °C and pulverized to a fine powder using a T-351 pulverizing machine (Rong Tsong Iron Co., Taichung, Taiwan). The nitrogen content was analyzed using an NC analyzer (Sumigraph NC-22F, Sumika Chemical Analysis Service, Osaka, Japan).

### Cell wall preparation

The cell wall residue (CWR) was prepared as described previously^76^ using the same dried powder sample as for N content analysis. The powder sample was further pulverized to a finer powder (TissueLyser, Qiagen, Hilden, Germany). The powder was sequentially extracted 20 times with methanol at 60 °C, five times with hexane at room temperature, and five times with distilled water at 60 °C. The residue was freeze-dried to obtain the CWR.

### Chemical analyses of the cell wall

Lignin content was determined using a thioglycolic acid lignin method^77^. The ultraviolet absorbance of thioglycolic acid lignin was measured at 280 nm using an SH-1000 lab microplate reader (Corona Electric Co., Ltd., Ibaraki, Japan). Analytical thioacidolysis was performed as previously described^76^. The released lignin monomers were derivatized with *N*,*O*-bis(trimethylsilyl) acetamide and quantified by gas chromatography-mass spectrometry (GC–MS) (GCMS-QP 2010 Ultra, Shimadzu, Kyoto, Japan) using 4,4’-ethylenebisphenol as an internal standard^78^. Cell wall-bound *p*CA and FA were released by a mild-alkaline treatment and quantified by GC–MS, as previously described^79^. Approximately 10-mg aliquots of CWR samples were placed in tubes and mixed with 1 M NaOH (1.5 mL), then degassed with oxygen-free N_2_. The suspension was incubated at 25 °C for 24 h with gentle shaking. The suspension was centrifuged and the supernatant was transferred to a new tube, *o*-coumaric acid was added as an internal standard, then subjected to GC–MS analysis. Glycosyl residue composition was determined by alditol acetate methods^80^. Approximately 15 mg of CWR samples were suspended in a 540 µL thermostable ɑ-amylase (Megazyme, Bray, Ireland) solution that was diluted 30-fold with 100 mM sodium acetate buffer, then incubated at 100 °C for 12 min. The suspension was cooled to about 40 °C, added with 18 µL of amyloglucosidase (Megazyme), and incubated at 50 °C for 30 min. The residue was washed twice with distilled water and twice with methanol, then dried under vacuum for 2 h. An aliquot of the destarched CWR was subjected to a hydrolysis with 2 M TFA at 100 °C for 5 h. The released monosaccharides were reduced with sodium borohydride and converted to alditol acetates using acetic anhydride. The alditol acetates were quantified using GC–MS (GCMS-QP 2010 Plus, Shimadzu) equipped with SP-2330 column (30 m × 0.25 mm × 0.2 µm film thickness, SUPELCO, Bellefonte, PA, USA) using *myo*-inositol as an internal standard. The residues left after hydrolysis were used to quantify the crystalline cellulose. The residues were added to the Updegraff reagent^81^ and heated at 100 °C for 30 min, washed twice with distilled water and acetone, and then hydrolyzed with 72% sulfuric acid^82^ at 30 °C for 1 h. The released glucose was quantified using the Glucose CII test kit (Wako Pure Chemicals Industries, Osaka, Japan). Uronic acid content was measured by the *m*-hydroxydiphenyl assay^83^ using galacturonic acid as a standard. The absorbance was measured at 520 nm using an SH-1200 lab microplate reader (Corona Electric Co., Ltd.). The calcium content was determined by atomic absorption spectroscopy (AA-6200, Shimadzu) after digesting the CWR with nitric and sulfuric acids. Polysaccharides in CWR were methylated using NaOH and methyl iodide^84^. To the CWR (10 mg) suspended in 600 µL DMSO, 600 µL of NaOH-DMSO suspension, and 300 µL of methyl iodide were added. The suspension was sonicated for 5 min and incubated at ambient temperature for 3.5 h with stirring. After adding 900 µL of distilled water, the per-*O*-methylated CWR was rinsed with chloroform, air-dried, and then subjected to hydrolysis of matrix polysaccharides with 4 M TFA at 120 °C for 1 h. Partially methylated monosaccharides released into the supernatant were converted to alditol acetates and analyzed by GC–MS (GCMS-QP 2010 Plus) equipped with SP-2330 column (SUPELCO). The molar ratio of the peaks was calculated using the peak area and the effective carbon-response factors^85^.

### 2D HSQC NMR

Finely ball-milled CWR samples (~ 60 mg) were swelled in DMSO-*d*_6_/pyridine-*d*_5_ (4:1, v/v) for the gel-state whole cell wall NMR analysis, as described previously^43,44^. NMR spectra were acquired on a Bruker Avance III 800US system (800 MHz, Bruker Biospin, Billerica, MA, USA) fitted with a cryogenically cooled 5-mm TCI gradient probe (Bruker Biospin). Adiabatic 2D HSQC NMR experiments were conducted using the standard implementation (‘hsqcetgpsp.3’) with parameters described in the literature^43,44^. Data processing and analysis were performed using the Bruker TopSpin 4.1 software (Bruker Biospin) as described previously^86–88^. For volume integration analysis, the aromatic contour signals from lignin and hydroxycinnamates (C2–H2 correlations from **G** and **F**; C2–H2/C6–H6 correlations from **S** and **P**; and C2ʹ–H2ʹ/C6ʹ–H6ʹ correlations from **T**, **S**, **P**, and **T** integrals were logically halved) and polysaccharide anomeric signals listed in Fig. 2 were manually integrated and each signal was normalized based on the sum of the **S** and **G** lignin aromatic signals (**½S**_**2/6**_** + G**_**2**_)^88^.

### Gene expression analyses

RNA-sequencing data of sorghum seedlings from our previous study (accession number DRA010070)^47^ were used for the analysis of the change in transcriptome induced within 3 or 6 d after low N treatment. For RT-qPCR analysis, total RNA was extracted from fully expanded uppermost leaves of 6-week-old (3 weeks after low-N treatments) seedlings using the Total RNA Extraction Kit Mini (Plant) (RBC Bioscience, New Taipei City, Taiwan) according to the manufacturer’s instructions with on-column deoxyribonuclease treatment. First-strand cDNA was synthesized as described^47^ using ReverTraAce DNA polymerase (Toyobo, Osaka, Japan). Quantitative PCR analyses were performed as previously described^47^ using KDO SYBR qPCR mix (Toyobo) on TaKaRa PCR Thermal Cycler Dice Real Time System II TP 960 (Takara Bio, Shiga, Japan). The primers used for PCR are listed in Supplemental Table S1. The amounts of the targets were estimated by ΔΔCt method using the PP2A gene as the internal control^89^.

### Immunofluorescence and immunogold labeling analyses

The aboveground parts of 6-week-old sorghum seedlings were sampled and fixed in 4% paraformaldehyde in 50 mM phosphate buffer (pH 7.4). Samples were dehydrated in a graded ethanol series (30%, 50%, 70%, 80%, 90%, 95%, and 99.5%) and then embedded in LR White Hard Grade (London Resin Co. Ltd., Reading, UK). For immunofluorescence histochemistry, transverse sections 0.5 μm thick were cut using an Ultracut E microtome (Reichert-Jung, Vienna, Austria). The sections were blocked with 3% (w/v) skim milk in Tris-buffered saline (TBS; 20 mM Tris–HCl, 154 mM NaCl, pH 8.2) for 30 min and incubated at 4 °C for one d with either of the following monoclonal antibodies: anti-xylan (LM10, PlantProbes), anti-arabinoxylan (LM11, PlantProbes), anti-xyloglucan (LM15, PlantProbes), anti-glucuronoxylan (LM28, PlantProbes), anti-MLG (400-3, Biosupplies, Australia), and anti-callose (400-2, Biosupplies Australia). The sections were washed three times with TBS and incubated at 35 °C for 2 h with either goat anti-rat immunoglobulin (IgG) conjugated to Alexa Fluor 488 (Molecular Probes, Oregon, USA) or with goat anti-mouse IgG conjugated to Alexa Fluor 488 (Molecular Probes) for PlantProbes antibodies or Biosupplies antibodies, respectively. The slides were washed three times with TBS and once with deionized water. After drying, the sections were mounted using ProLong Diamond (Thermo Fisher Scientific, Oregon, USA), and observed under a fluorescence microscope (BX50 with BX-FLA fluorescent light attachment; Olympus, Tokyo, Japan) using a U-MWIB3 filter set (Olympus; 460–490 nm excitation, 515 nm long-pass emission). For immunogold labeling, ultrathin transverse sections were cut using an Ultracut E microtome and transferred to 300-mesh nickel grids (Nisshin EM Co. Ltd., Tokyo, Japan). The grids were blocked in 20 µL of 0.1% (w/v) sodium azide in TBS containing 1% BSA at room temperature for 30 min. They were then incubated with 20 µL of LM11 and MLG at 4 °C for one day. After washing three times with TBS, the grids were incubated at 35 °C for 2 h either with 10 nm colloidal gold conjugated secondary antibody goat anti-rat IgG or anti-mouse IgG (BBI solutions, Crumlin, UK) for LM11 or MLG, respectively. The grids were washed three times with TBS and once with deionized water, stained with 2% uranyl acetate for 10 min, and then washed again with deionized water. The grids were observed under a JEM-1400 transmission electron microscope (JEOL, Tokyo, Japan) operated at 100 kV.

## Supplementary Information

Supplementary Information.

## Data Availability

All data necessary to evaluate the conclusions in this study are included in the published paper and its Supplementary Information file. Additional data, if required, will be made available by the corresponding authors upon request.
