# Supplementary material for: Nitrogen deficiency results in changes to cell wall composition of sorghum seedlings
Source: Sci Rep. 2021 Dec 2;11:23309. doi: 10.1038/s41598-021-02570-y (PMC8640004; doi:10.1038/s41598-021-02570-y)
Supplement: Supplementary file 1 — Supplementary Information. [file 41598_2021_2570_MOESM1_ESM.pdf]

Nitrogen deficiency results in changes to cell wall composition of sorghum seedlings

Reza Ramdan Rivai, Takuji Miyamoto, Tatsuya Awano, Rie Takada, Yuki Tobimatsu, Toshiaki Umezawa, Masaru Kobayashi

Gene name	Gene ID	Forward primer (5′→3′)	Reverse primer (5′→3′)
CESA	Sb02g010110	TAATGTTGCCAGCCTGTGGT	GAACACAGCAAAGAGGTGCG
CSL	Sb07g004110	CTCTCCTACAACTGGCCGTG	GCCGCCTCACCATATCATCA
Endo-1,4-β glucanase	Sb01g008860	CAAGTTTGCCAGGTCACAGC	GCGCTTCGGGTACTTGTTTC
Pectate lyase	Sb08g004905	CCACCATGGCCAGAGGTATG	CTCAGGAGCTTGGATAGCGG
Glucan-1,3-β-glucosidase	Sb03g045460	GGCTCACCTACACCAACCTG	GCCCCTGGTTGTACTTCCTC
GAUT	Sb01g012060	GCCGAGAGAGAAAAGCCGAG	TCAGGCGAGGTAAATGGTGG
EXP	Sb03g038290	CAGTTCTAGCACGCCCCTC	AGGAAATGCCTAAGCGGGTG
XTH	Sb10g028570	GATAAGTACCGCTTCCCGCA	CAAGTCATCATGCACACGGC
GSL	Sb04g038510	CTTATCAAACTGCCGCCGTG	TGCGTCTCGAGAATCGACTG
LAC	Sb03g039970	CCTTCCTCAGCACAAGGAGC	CGAGGTTCCCGGTTGATCTC
RG-I lyase	Sb07g024560	AGGGAGAACGCGATAGCAAG	CAATCCCTGAAACGGGCTCT
PAL	Sb04g026520	CCAAAGTACAGCGGCTCAAG	CAAGAACATGCGCATTGCAG
4CL/ Bmr2	Sb04g005210	CATCTCCAAGCAGGTGGTGT	ATTGCACGTAACAAGGCACG
CAD/ Bmr6	Sb04g005950	TACCCTATGGTCCCTGGGC	GCCGTCAGTGTAGACATCGT
C3'H	Sb09g024210	ACCTTCTGCACCACTTCGAG	AGGCACCTCACATCTCAACG
F5H/ CAld5H	Sb01g017270	ATGGCGGAGATGATGCACAG	CGTCTCCTTGATGACGCACT
COMT/ CAldOMT/ Bmr12	Sb07g003860	TTAATGGCCTAGCCTGCCTC	CGCAGAGACAATTCGACAGC
PP2A	XM_002453490	AACCCGCAAAACCCCAGACTA	TACAGGTCGGGCTCATGGAAC

Supplemental Table S1. List of primers used for reverse transcription-quantitative PCR analysis
